# Exploring Consent to Use Real-World Data in Lung Cancer Radiotherapy: Decision of a Citizens’ Jury for an ‘Informed Opt-Out’ Approach

**DOI:** 10.1007/s10728-025-00510-9

**Published:** 2025-02-10

**Authors:** Arbaz Kapadi, Hannah Turner-Uaandja, Rebecca Holley, Kate Wicks, Leila Hamrang, Brian Turner, Tjeerd van Staa, Catherine Bowden, Annie Keane, Gareth Price, Corinne Faivre-Finn, David French, Caroline Sanders, Søren Holm, Sarah Devaney

**Affiliations:** 1 Manchester Centre for Health Psychology, Division of Psychology and Mental Health, School of Health Sciences, Faculty of Biology, Medicine and Health, The University of Manchester, Manchester, UK; 2 Vocal, Research and Innovation Division, Manchester University NHS Foundation Trust, Manchester, UK; 3 Department of Radiotherapy Related Research, Division of Cancer Sciences, School of Medical Sciences, Faculty of Biology, Medicine and Health, The University of Manchester, Manchester, UK; 4 RAPID-RT PPI Advisory Group, Manchester, UK; 5 Centre for Health Informatics and Health Data Research UK North, Division of Informatics, Imaging and Data Science, School of Health Sciences, The University of Manchester, Manchester, UK; 6 Centre for Social Ethics and Policy, Division of Law, School of Social Sciences, The Faculty of Humanities, The University of Manchester, Manchester, UK; 7 Department of Clinical Oncology, The Christie NHS Foundation Trust, Manchester, UK; 8 Centre for Primary Care and Health Services Research, NIHR Greater Manchester Patient Safety Research Collaboration, Division of Population Health, Health Services Research and Primary Care, School of Health Sciences, Faculty of Biology, Medicine and Health, The University of Manchester, Manchester, UK

**Keywords:** Real-world data, Citizens’ jury, Patient consent, Informed opt-out

## Abstract

An emerging approach to complement randomised controlled trial (RCT) data in the development of radiotherapy treatments is to use routinely collected ‘real-world’ data (RWD). RWD is the data collected as standard-of-care about all patients during their usual cancer care pathway. Given the nature of this data, important questions remain about the permissibility and acceptability of using RWD in routine practice. We involved and engaged with patients, carers and the public in a two-day citizens’ jury to understand their views and obtain decisions regarding two key issues: (1) preferred approaches to consent for the use of RWD within the context of patients receiving radiotherapy for lung cancer in RAPID-RT and (2) how RWD use should be best communicated to patients. Individual views were polled using questionnaires at various stages of the jury, whilst group discussion activities prompted further dialogue about the rationale behind choices of consent. Key decisions obtained from the jury include: (1) an opt-out approach to consent for the use of RWD; (2) the opt-out approach to consent should be informed. Furthermore, it was advised that information and communication regarding the consent process and use of RWD should be accessible, clear and available in a variety of formats. It is important that the consent process for patient data use is underpinned by principles of autonomy and transparency with clear channels of communication between those asking for and giving consent. Moreover, the process of seeking consent from patients should be proportionate to the risks presented from their participation.

## Introduction

Radiotherapy is one of the most important and cost-effective methods of treating cancer [1]. The success of radiotherapy treatment can be partly attributed to a rich history of technical and technological evolution, which has enabled the implementation of more precise and risk-adapted practices [2–3]. Traditionally, evidence from randomised controlled trials (RCTs), commonly used to establish cause and effect relationships between interventions and outcomes, has informed evaluation of changes in radiotherapy [3–5]. However, setting up RCTs can be resource and time-intensive, whilst certain patient groups remain under-represented such as patients from ethnic minority groups and patients living with co-morbidities [6–8]. An emerging approach to complement conventional RCT data is to use routinely collected ‘real-world’ data (RWD) to provide evidence of the clinical impact of changes in practice [2, 9]. This approach offers the opportunity to track changes through a series of iterative ‘rapid-learning’ cycles that are informed by quality improvement principles [3, 10–11].

The National Institute for Health and Care Research (NIHR)-funded RAPID-RT method-development programme [12] seeks to explore how the rapid-learning concept, which uses RWD, can provide evidence to optimise radiotherapy for patients with lung cancer. RWD is the data routinely collected as standard-of-care about all patients during their usual cancer care pathway [2, 13]. RAPID-RT is a prospective observational study in which the exemplar clinical change being evaluated is a change to the standard-of-care radiotherapy protocol delivered with curative intent for all patients living with stage I-III lung cancer [2]. The standard-of-care was changed in April 2023 when a radiotherapy dose limit for the top of the heart was introduced for all eligible patients treated at The Christie NHS Foundation Trust, Manchester, UK [12]. Rapid-learning involves the use of RWD to evaluate this change through comparison with the historic cohort treated with the previous, non-heart sparing, standard-of-care radiotherapy. The results from this evaluation are then used to inform the decision, by the clinical team, to further refine this change if required. Repeating this process over subsequent iterative cycles then allows the optimisation of the change in practice to give the best outcomes in the target patient cohort [2–3]. This considered, there remain important questions regarding the permissibility and acceptability of using RWD in approaches of this nature. In this article, we summarise the decisions obtained from a citizens’ jury held to determine the appropriate form of consent to allow the use of RWD in RAPID-RT and how this should be best communicated to patients. We also report on how these decisions have been implemented in RAPID-RT.

## Background

### Real-World Evidence

The National Institute for Health and Care Excellence (NICE) defines RWD as ‘data relating to patient health or experience or care delivery collected outside the context of a highly controlled clinical trial’ [14]. RWD can be structured or unstructured and includes, for example, data collated in patients’ electronic health records (EHRs), data derived from disease registries or data from other sources that can inform health status [14–15]. Unstructured RWD (e.g. free text data regarding clinical information) is likely to be more narrative and contain more contextual information about the patient background [16]. Structured RWD, on the other hand, are organised using a set of predefined clinical concepts (e.g. Read Codes) which may make it easier for formatting and processing [16]. Given RWD is routinely collected as standard-of-care about *all* patients, it has the potential to provide more representative and inclusive evidence than that collected in RCTs [17–18]. However, repurposing routinely collected data can be challenging with efforts influenced by ethical, legal, and technical complexities [19–20]. Compliance, such as maintaining data security for example, may be required under existing legal provisions.

### Obtaining Lawful Access to Health Data in RAPID-RT

Consent for the provision of treatment to patients is embedded at the core of the UK legal system [21]. In contrast, when accessing health data for research, while consent is a key element and can provide the legal gateway to accessing patient data, it is not always the primary mechanism which provides the legal basis for access [22–23]. The data being accessed in RAPID-RT is routinely collected throughout patients’ treatment. This data, linked from the hospital’s different information systems, is pseudo-anonymised before it leaves the treating hospital’s NHS computer network. This is then pushed into the RAPID-RT database held on a secure subnet of The University of Manchester computer network which is physically based in the treating hospital and secured from the wider university network. The pseudo-anonymisation key table is secured on the hospital network and is not accessible to anyone analysing data on the RAPID-RT database. Before any of the linked data is released from the RAPID-RT database for analysis by researchers, it is fully anonymised i.e. there is no record of allocation of anonymous IDs.

Under the law of confidentiality [24], the care team possesses legitimate reasons to access and anonymise the data and so would not be seen as breaching patient confidentiality. Equally, UK data protection law requires a lawful basis for the research processing of health data. Typically in this context, such basis is provided if processing is in the ‘public interest’, which applies here. Data that is no longer attributable to an identifiable individual is not personal data. Therefore, fully anonymised data lies outside of UK GDPR as well as the common law duty of confidentiality and as such explicit consent to access the data would not be legally required. In RAPID-RT, a legal basis is required for the care team to process the data to anonymise it, which is fulfilled in this context. In contrast, the RAPID-RT research team can access the anonymised data without such justification. However, as we set out later in this article, members of our citizen’s jury decided that RAPID-RT should implement an informed opt-out approach to consent for the study even though this is not strictly required in law.

### Consent in Health Research

There are several approaches to consent in relation to participation in health research. Traditionally, an explicit opt-in approach has been adopted, the outcome of which has been recorded in writing [25–26]. There is, however, growing acceptance that consent methods should reflect the relative risks and burdens of the proposed research [27–29]. Where an opt-out approach to consent is adopted, an individual is enrolled in a study unless they actively signal that they do not wish to be involved. Certain studies may also be designed where no additional consent is taken and where the individual is involved without being asked, such as studies which use a pre-existing, anonymised data set. In these types of studies, it is likely that guarantees of data anonymisation (through earlier de-identification measures) are in place.

In many countries, there is no legal impediment to the use of deidentified (or anonymised) data for research without the consent of the data subject, if the risks of reidentification are very low or remote [26, 30]. Separately to specific research studies, NHS England currently operates a general, national data opt-out mechanism for the collection and use of patient data [31]; this applies to the disclosure of confidential patient information for purposes beyond individual care and aligns with the common law duty of confidentiality. In broad terms, where people have opted out of sharing their data through the national data opt-out system, this will be respected unless there is a mandatory legal requirement or an overriding public interest for the data to be shared. For example, during the COVID-19 pandemic, the UK government issued a Control of Patient Information (COPI) notice which superseded the national data opt-out mechanism, allowing healthcare organisations and local authorities to process confidential patient information, regardless of its identifiability, for the purposes of responding to COVID-19 and associated public health risks [32–33].

### Patient and Public Involvement and Engagement (PPIE) in RAPID-RT

The two-day citizens’ jury considered in this article is one element of a number of PPIE approaches embedded across the RAPID-RT programme, which has had a commitment to PPIE since its inception. The programme collaborates with Vocal, a not-for-profit organisation hosted by Manchester University NHS Foundation Trust in partnership with The University of Manchester, whose aim is to bring people and health research together for mutual benefit. Before applying for funding for RAPID-RT, its acceptability, feasibility and design were shaped by extensive discussions with several patient discussion groups over three years, including the Black, Asian and Minority Ethnic Research Advisory Group (BRAG) and Get Vocal on Cancer Network. A community sandpit event was also developed by Vocal in partnership with the Greater Manchester (GM) Black and Minority Ethnic Network, with patients, carers and advocates contributing to these considerations. Furthermore, there are two patient co-applicants with lived experience of radiotherapy on the programme team, who have helped to shape programme development since the beginning. RAPID-RT is also supported by a patient advisory group (PAG) which meets biannually to discuss, reflect and advise on the programme.

### **Citizens’ Jury Design**

This article reports on the collective decisions made at a two-day citizens’ jury held in May 2022 with members of the public, carers and people living with cancer on the appropriate consent and patient information approaches which should be implemented for the use of anonymised RWD in RAPID-RT. A citizens’ jury is a deliberative dialogue process which involves a group of people (‘jurors’) coming together over a period of time to further their knowledge and understanding of a particular issue and associated social and ethical factors [34]. Given enough time, opportunity and support, members of the public can arrive at decisions (‘verdicts’) about complex matters and justify to decision makers what an informed public wants and why they want it [16, 34]. In this manner, a citizens’ jury encourages discussion and opinion sharing before moving to decision-making.

The design of our citizens’ jury followed guidance written by the Jefferson Centre [34], the developers of the method, with practical support provided by Vocal. The key questions presented to the jury were informed by a shorter, one-day citizens’ jury held in February 2020 with ten radiotherapy patients and carers [12]. The mini-jury was sampled from the Get Vocal contact database to represent different GM communities. The mini-jury considered the acceptability of different methods of recruiting and consenting patients to participate in RAPID-RT. A key jury conclusion here was that the use of pragmatic consent models are acceptable in this context. This decision directly informed the methodological design of both the clinical study and the questions used to guide the two-day citizens’ jury examined in this article. Jurors were asked to consider the following questions over the two-day citizens’ jury:

### Question 1: Consent

What is the most appropriate way for patients to consent to their anonymised data being used in the RAPID-RT study?

### Question 2: Patient Communication

How do you think information about the RAPID-RT study should be delivered to our patients?

### Recruitment to the Jury

The organising team focused recruitment efforts on two main criteria: (1) the ethnic diversity of the GM area and (2) the prevalence of lung cancer within socioeconomic groups. In England, lung cancer particularly affects older populations, with incidence rising from age 40–44 and peaking between 79 and 89 [35]. Incidence rates are disproportionately higher for adults in the most economically deprived quintiles, associated with higher smoking rates, poorer lifestyles and decreased access to care [35]. Additionally it was noted that racialised individuals are more likely to live in areas of deprivation [36]. Vocal were responsible for the recruitment of jurors. To ensure widespread engagement, advertisements were circulated through existing Vocal’s PPIE Networks. The invite targeted patients with direct experience of radiotherapy, carers of patients with cancer who had received radiotherapy and members of the public with an interest in healthcare research. Targeted recruitment campaigns within areas of deprivation and amongst minority ethnic community networks in GM, an area covered by ten local authorities, was prioritised. According to the 2019 Index of Multiple Deprivation [37], five GM districts rank in the most deprived decile, amongst a list of thirty most deprived local authorities in England. Manchester, a city in the GM district, is the sixth most deprived local authority in England, with 43% of neighbourhoods identified as highly deprived [37]. Recruitment of patients with lung cancer who had experience of radiotherapy was challenging due to the prevalence of lung cancer within older populations and the relatively low 10-year survival rate of 10% [38]. The research team also recognised the significant time commitment required to join the jury. As the citizens’ jury was a PPIE initiative, NHS Research Ethics Committee approval for its conduct was not required.

The in-person jury convened over two days with a total of 24 jurors participating. One person was unable to attend day 2 of the citizens’ jury, leaving 23 participants. Guidance recommends 12–24 people per citizens’ jury to allow for effective deliberation [34]. Jurors included individuals with either (a) direct experience of treatment or radiotherapy for lung cancer; (b) experience of caring for someone who had received radiotherapy or treatment for lung cancer; or (c) members of the community with an interest in health research (See Table 1). 71% of participants were aged 50 or over and 29% of participants were non-white British or Irish.

**Table 1:** Demographics of jurors attending the RAPID-RT citizens’ jury

Criteria	No. of Jurors
Gender	
Male	7 (29%)
Female	17 (71%)
Age	
< 29	2 (8%)
30–49	5 (21%)
50–69	14 (58%)
70+	3 (13%)
Ethnicity	
White British	15 (63%)
Black African/Caribbean	2 (8%)
British Asian Pakistani	3 (13%)
Mixed Asian and White	1 (4%)
Chinese	1 (4%)
White Irish	2 (8%)
Location	
Greater Manchester	20 (83%)
Outside of Greater Manchester	4 (17%)

### Citizens’ Jury Programme

A design feature of a citizens’ jury is the combination of organised activities that include topic-related presentations, group discussion sessions and polling activities [34]. Through this set of activities, jurors develop their understanding, obtain answers for any questions they may have, whilst also seeing other jurors’ views in real-time. A citizens’ jury design group consisting of RAPID-RT researchers and patient co-applicants, Vocal staff and a member of BRAG developed the RAPID-RT citizens’ jury programme. An external academic and a member of BRAG met separately before the jury to review the programme and ensure that the information presented was balanced and neutral.

#### Presentations

Presentations were delivered by two groups: (1) ‘Citizens’ friends’; and (2) ‘Expert witnesses’ (See Table 2). Citizens’ friends were members of the RAPID-RT study team who were responsible for clarifying details about RAPID-RT and answering study-specific questions. In contrast, expert witnesses were impartial stakeholders, who had been invited to describe the different elements of data-sharing and consent processes without enforcing a particular viewpoint. Six expert witnesses were, at the time, in professional posts (academic, clinical, regulatory), whilst one expert witness was a patient who had undergone radiotherapy for lung cancer; the latter’s talk reflected on the expert witness’s experience of the consent process in a separate data-related study. The difference between expert witnesses and citizens’ friends was explained to jurors at the start of the jury.

**Table 2:** Presentations given to jurors during the RAPID-RT citizens’ jury

Day 1	
Content of presentation	Purpose of presentation
**Talk 1: Introduction to RAPID-RT: Lung cancer, rapid-learning, RWD and problem statement** ***RAPID-RT Study Team***	
• What is radiotherapy and how changes to radiotherapy treatment are made • Purpose and design of RAPID-RT	• Understanding of rapid-learning and RWD • Understand how RWD will be used in the treatment of lung cancer
**Talk 2: How do we design research studies?** ***RAPID-RT Study Team***	
• What research is and how studies are designed • The particularities of RAPID-RT	• Advantages and disadvantages to the different ways of setting up research studies
**Talk 3: How does consent work?*Expert in Bioethics***, ***The University of Newcastle***	
• The history of healthcare research and how it has informed current modern-day laws • The different ways in which consent can be given and patients’ rights in research	• The advantages and disadvantages of each approach to consent
**Talk 4: Legal approvals needed for research studies involving people and their data** ***Expert in Research Development***, ***Health Research Authority (HRA)***	
• The different bodies that exist to regulate research studies • The law and how it relates to healthcare research • The law and how it applies to RAPID-RT	• The purpose of consent • The different criteria that underpin taking consent from patients
**Talk 5: Using data in research*Expert in Health Informatics***, ***The University of Manchester***	
• Details about electronic health records • Advantages and disadvantages of using health records for research	• Understand how health records are used in a healthcare setting • The value of data for research
**Day 2**	
**Content of presentation**	**Purpose of presentation**
**Talk 6: Patient experience of the consent process in the CONCORDE Study** ***Expert Patient***	
• Describe personal experience of the consent process in the CONCORDE study*	• What written consent looks like in a real study • Factors that may influence consent giving
**Talk 7 & 8: Introduction to the WHEAT Study** ***WHEAT Trial Study Team***	
• Introduced a research study called WHEAT^*^ that used opt-out consent • Advantages and disadvantages of opt-out consent (as reported in the study)	• Understand the complexities of the opt-out process

Notes

*CONCORDE Study [39]: A clinical study trial to find the best dose of certain targeted drugs to give alongside radiotherapy for people with non-small cell lung cancer (NSCLC)

*WHEAT Study [40]: The WithHolding Enteral Feeds Around Transfusion (WHEAT) study is a randomised pilot trial evaluating the feasibility of comparing two care pathways, withholding and continuing feeds around transfusion for babies born before 30 gestational weeks

#### Group Discussions

For group discussion, jurors were divided into four groups of equivalent numbers and remained in these groups over the two-day jury period. Some jurors were split between the groups to ensure their support needs were met, whilst other jurors were given the option to choose where to sit. Group discussion took place after a presentation had been delivered, allowing jurors to reflect on the key issues that had been raised, along with their own views of consent approaches. Approximately, 160 min in total (day 1: 60 min; day 2: 100 min) was allotted for group deliberations. Group discussions were audio-recorded for purposes of summarising the key discussion points. In addition, each group had a designated facilitator and separate note-taker. Two of four facilitators were patient co-applicants on the RAPID-RT study team whilst the other two facilitators were Vocal team members. Similarly, two of four note-takers were members of the RAPID-RT research team and two were Vocal team members. Pre-jury meetings had taken place with facilitators and note-takers to clarify roles and expectations. Both groups were given respective guides on how to encourage group discussion and ensure depth and quality of note-taking. The purpose of taking notes (in addition to recording discussions) was to allow facilitators to share deliberations back to the entire jury after the end of each group discussion activity. During this sharing exercise, there were several opportunities for jurors to seek clarification on points of fact from citizens’ friends and expert witnesses.

#### Polling

The citizens’ jury design group had developed polling activities to be carried out during the jury. Jurors reflected on various consent options and the delivery of study information through polling questionnaires at three different time points across the two days: (1) start of the jury day one (P1); (2) close of jury day one (P2); and (3) close of jury day two (P3). Jurors were asked to reflect on the same two jury questions (question 1: consent; question 2: patient communication) at each polling stage. Jurors were asked to circle their preferences for each of the consent and information delivery options available rather than choosing a single best option (See Appendix 1). For the first question, jurors had four consent options to consider which had been tested within the mini-jury: *fully informed opt-in*, *simplified opt-in*, *opt-out*, *or no additional consent needed*. For the second question, jurors had seven communication options to consider: *in person by a healthcare professional (HCP)*, *via participant information sheet (PIS)*, *via QR code*, *via poster*, *via flyer*, *short online film*, *or recorded telephone message*. A six-point Likert scale was used for each question: *Strongly Agree (SA)*, *Agree (A)*, *Disagree (D)*, *Strongly Disagree (SD)*, *Do Not Know (DNK)*, *Do Not Understand Question (DNU).*

### Recording of Deliberations, Decisions and Advice

In relation to questions 1 and 2, graphs were created on a rolling basis throughout the citizen’s jury to display results of all polling in clear pictorial form. These were presented for a final time to jurors at the end of day 2 after all polling had been conducted to provide an indication of how the jury responded to the key questions posed. Recorded group discussions were transcribed by respective note-takers and shared with the author (AK) responsible for examining the results. The transcribed notes were reviewed alongside the results of polling, with a descriptive approach taken whereby key discussion points that specifically revolved around preferred consent and communication choices were identified and explored in more detail [41]. Views pertaining to these choices, across groups, were linked together into ‘summaries’ (deliberations and decisions) to illustrate what conclusions were arrived at, and the reasons articulated for their suggestions; this was undertaken at the whole jury level.

### **Citizen’s Jury Deliberations, Decisions and Advice**

#### Decision 1: An Opt-Out Approach to Consent for the Use of RWD

**Fig. 1:**
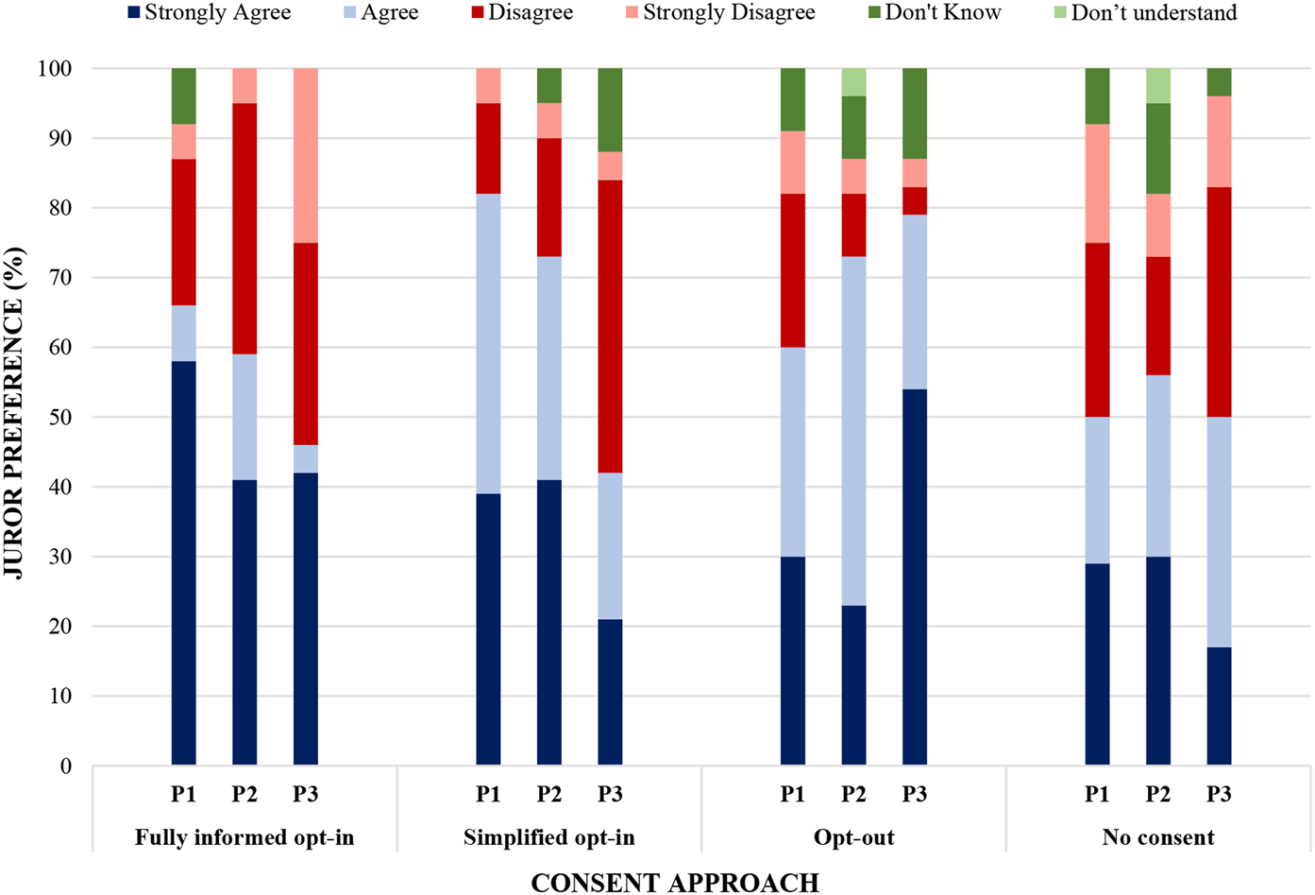
Poll responses to ‘Q1.What is the most appropriate way for patients to consent to their anonymised data being used in the RAPID-RT study?’. This graph shows how jurors’ preferences of consent approaches evolved over the course of the jury. P3 shows the results from the last poll conducted at the end of day 2 for each consent option: this poll shows greatest support (as indicated by combining SA and A scores) for opt-out (79%) followed in order by no consent (50%), fully informed opt-in (46%) and simplified opt-in (42%)

Poll results indicated the jury’s preference for an opt-out approach in RAPID-RT as indicated by combined SA and A scores (79%) at the final polling point (Poll 3) (See Fig. 1). Jurors’ preferences evolved over the course of the jury; initially, greatest support was given for fully informed (67% SA/A) and simplified opt-in approaches to consent (83% SA/A). In contrast, at the end of the jury, the percentage of jurors either strongly agreeing or agreeing that fully informed or simplified consent were more appropriate had fallen to 46% and 42% respectively. As part of the polling exercise, jurors were also asked for their views on having no additional consent process for the use of RWD. Poll results indicated relatively moderate support for no consent, which remained consistent across both days: Poll 1: 50% SA/A; Poll 2: 57% SA/A; Poll 3: 50% SA/A.

Several reasons were given by jurors for their preference for an opt-out approach. Groups discussed how opt-out approaches could lead to greater access to RWD by virtue of engaging more patients, which, to them, could also result in a more representative population [17, 42–43]. In addition, jurors described an opt-out approach to consent as normalising participation and being reflective of an altruistic obligation to share data for the benefit of patients through the improvement of health services. In contrast, opt-in approaches, despite being what most jurors had previous experience and familiarity with, were described as requiring more time and effort (e.g. to read lengthy information sheets and return consent forms), which could impact negatively on participation rates. Constraints of time and accessibility have been associated with opt-in approaches to consent, where participation has sometimes been impacted [44–45].

The fact that the standard-of-care treatment is measured as part of RAPID-RT (as opposed to it changing or affecting any care provided to patients) and that RWD is anonymised appeared a principal factor in jurors’ discussions about consent. Jurors subsequently categorised the use of anonymised RWD in the study as ‘low-risk’ which appeared to result in support for the use of this data, if managed appropriately. Existing research shows that the public is generally supportive of data-sharing when it is seen to have a public benefit and low-risk is attached to their involvement [29–30]. Where studies are considered low-risk and are using secondary health data that has already been collected for another purpose, the need to obtain consent at all has been disputed [25, 43, 46]. It is, for example, on this basis that NHS England proceeds with a general opt-out approach to patient data-sharing. This further reiterates how differences exist between public expectations of how personal data may be processed and what is permissible by law which can allow for no consent to be obtained in circumstances where risks of reidentification remain low [19, 23]. Whilst members of the jury considered the use of RWD as low-risk, greater support for opt-out approaches compared to no consent (as observed in the jury results) may be explained by the perceived absence of information and choice within non-consent approaches. Jurors in our citizens’ jury felt strongly about the need for information and choice, even if there was minimal risk attached.

#### Decision 2: The Opt-Out Approach to Consent Should be ‘Informed’

Jurors were open to sharing their data in RAPID-RT if this were supported with clear information about how their data would be used. In this respect, participants identified ‘informed opt-out’ approach as the most suitable mechanism for consent-giving towards the use of RWD. The request for clear information built on jurors’ suspicions of wider data-sharing practices, informed by well-documented examples of poor data management in other contexts. For example, several jurors raised the Facebook-Cambridge Analytica data scandal during discussions. In this case, a review found that personal data belonging to millions of Facebook social media users had been collected for the purposes of political advertising [47]. Furthermore, jurors raised themes of mistrust, misinformation, and misunderstanding as characteristic of data sharing generally and as a result, only approved of data use for direct patient benefit. They perceived commercial research as failing to uphold this aim.

In jurors’ views, clarifying details of RAPID-RT to patients through provision of relevant, clear and understandable information not only offered reassurances about data use, but further implied a sign of respect towards them and the use of their data. Jurors characterised ‘informed choice’ as strengthening trust and transparency between patients and professionals through the enhancement of participant autonomy and control [40, 48]. Pertinently, then, jurors’ preference for opt-out over no additional consent may indicate how they prioritise values of choice, respect, and transparency ahead of, or irrespective of, how their data might be used [26].

The quality of supporting information provided with opt-out approaches has been debated within the literature. Several studies have reported how participants have often received insufficient information regarding opt-out approaches, which affected their capacity to make an informed decision about participation [29–30, 49]. Moreover, an informed opt-out consent mechanism may, for some, resonate with the design of a simplified opt-in consent procedure. Both opt-in and opt-out procedures require individuals to be adequately informed about the research, data protection and data governance. However, crucially, in an opt-out approach, an individual is presumed to consent if they do not actively refuse the use of their health data. In an opt-in approach, an individual is required to provide verbal or written confirmation for their data to be used. Consequently, key feedback received from members of the jury suggests how an opt-out approach should not necessarily mean that the consent process is less informative [40]. Rather, as we set out in the next section, providing reassurances of protection from risk through clear information channels can increase participant support for opt-out models [19].

### *Advice: Information and Communication Regarding the Consent Process and Use of RWD Should be Accessible, Clear and Available in a Variety of Formats*

**Fig. 2:**
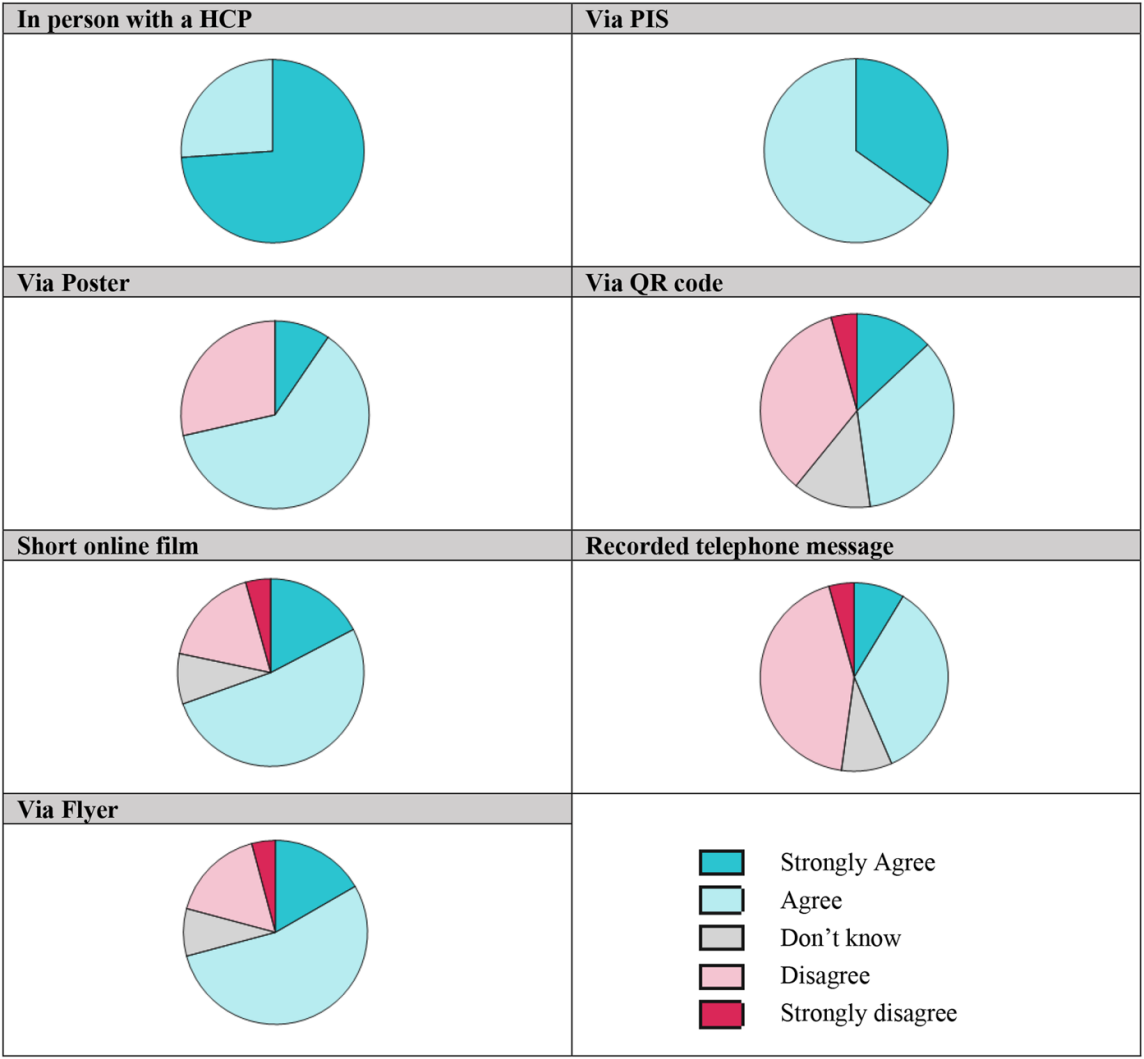
Final juror preferences (Poll 3) to ‘Q2. How do you think information about the RAPID-RT study should be delivered to our patients?’ Strongest preference was shown for in person communication with a HCP and via a PIS (100% agreement across jurors (SA and A responses combined). More jurors strongly agreed for in person communication (74%) compared to information via PIS (35%). SA/A responses for other methods with significant support include Via Poster (71% SA/A); Via Flyer (71% SA/A); Short Online Film (70% SA/A). Greatest disagreement (SD and D combined) was shown for information to be delivered via QR codes (39%) and recorded telephone messages (48%)

There was overwhelming agreement amongst jury members that multiple methods should be used to disseminate details of how RWD will be used in RAPID-RT (See Fig. 2). Support for a consultation with a HCP and a study PIS were the most preferred methods (> 83% SA/A); between these two methods, a consultation with a HCP (74% SA) was recognised as most optimum followed by a study PIS (35% SA). Verbal, in-person communication was described as offering patients greater confidence and security; information was being directly shared from a medical expert and patients also had the opportunity for any questions to be answered. However, jurors also discussed the need for an additional written form of documentation as this provided a physical data trail which could be helpful if any legal issues were to arise.

Regarding other methods of delivering study information, strong support was shown for study information to be delivered via flyers (71% SA/A), posters (71% SA/A) and a short online film (70% SA/A). This support was largely characterised through jurors agreeing rather than strongly agreeing as observed in the case of a consultation with an HCP. There appeared least appetite for information to be delivered using QR codes (48% SA/A) and recorded telephone messages (43% SA/A). Furthermore, the latter two methods recorded a higher percentage of negative responses: QR code (39% D/SD), recorded telephone message (48% D/SD). It must be noted that jurors strongly disagreeing to any single method was exceptionally low at the final poll point: only one juror was recorded as strongly disagreeing with information via a flyer, via a QR code, via a short online film, and via a recorded telephone message.

Using multiple methods to disseminate study information was recognised by jurors as a way of reaching a wider audience, improving inclusivity (through the ability to tailor communication to different patient groups) and representation of patients. Visual methods (such as a flyer, poster, and short online film) were considered likely to be more memorable. The accessibility of digital methods was viewed as having both positives and negatives. For example, some jurors spoke positively about the ease of using QR codes, whereas most jurors saw them as creating barriers for involvement and alienating certain groups such as older people, those without smartphone access, and less technologically proficient individuals. Jurors also criticised lengthy PISs with often complex language for creating barriers to engagement and leaving some patients unintentionally excluded. Clear and easily understandable information, written using lay terminology, was recognised as an important mechanism for addressing inclusivity. To overcome communication difficulties, jurors suggested greater patient involvement in the design of study materials. The involvement of patients in the design of informational material can help aid accessibility in several ways, which include for example, highlighting key areas that are likely of importance to participants, ensuring content is relevant and presenting information using clear, non-discriminatory language [50–53].

The timing of discussing consent for use of data with patients was further considered crucial by jurors given the emotive context of a recent cancer diagnosis. Jurors were notably concerned about being asked to discuss use of data in the study at such a challenging time. However, although timing was important, there appeared to be no consensus regarding the right time to discuss participation and consent. Rather, discussing participation in a study and its approach to consent should be context-dependent and individual to the patient, necessitating a level of flexibility on the part of the healthcare team. An overwhelming observation from the discussions that took place and results of polling was the need to communicate effectively the change being introduced to patients (the use of RWD in this instance) as this determines how likely they are to provide consent. The strong preference for a consultation with a HCP is perhaps unsurprising, then, given jurors expressed strong views of clearly understanding what is taking place in the study. In-person interaction is critical to uphold trust between HCPs and patient and provide a sense of security over how data might be used. Through this, trust and respect are upheld, and patients recognise the value of what is being implemented and how their participation can contribute [19].

### **Implications for Research and Practice**

The decision of the jury for an informed opt-out approach to consent for the use of RWD is consistent with the narrative of using a consent approach that is proportionate to the study risks [27–30]. Normatively idealised consent practices may not always be desired, or maybe even ill-suited for what is proposed [40, 54]. For example, the use of RWD in RAPID-RT could be legally approached through a non-consent approach given this data is routinely being collected (with no additional effect on patient and their care) and risks of reidentification are very low given the measures in place to handle RWD [25, 46, 55]. Additionally, the form used to consent patients for their radiotherapy has a statement included “I agree that information collected during my treatment, including images and my health records may be used for education, audit and research. All information will be anonymised. I am aware I can withdraw consent at any time” [56]. This considered, jurors still decided upon an informed opt-out approach for inclusion of their data specifically within RAPID-RT. Likening opt-out approaches to a form of altruism and public duty by jurors resonates with a view of research as being a normative good that compels people to get involved [29, 54, 57]. Additionally, the process through which patients are able to opt-out of data use using the radiotherapy consent form is unclear.

Responses from jurors also reveal how people place emphasis on values of autonomy, trust, and respect in relation to sharing their health data. Jurors’ willingness to share data and support for opt-out was still partly dependent on effective communication that can help improve inclusivity, accessibility, and trust. Feedback from the citizens’ jury echoed principles set out by Knowles and colleagues [58] regarding transparency measures when using patient health data. The authors highlight three key types of transparency: (1) informational transparency – information around what data is collected is made understandable to patients whose data is being used; (2) participatory transparency – patients are given the opportunity to be involved in decisions around data access and mobilisation; 3) accountability transparency – patients are informed about the outcomes of using this data. To secure cooperation, patients need to have the confidence that their data will be treated in a manner that adheres to the above principles, and where possible, this may extend beyond compliance laid down in a legal framework [19, 54].

## Strengths and Limitations

Using a citizens’ jury to elicit advice on which approaches to adopt for the use of RWD in RAPID-RT mean that the study is informed in a way that patients and members of the public may deem acceptable. The citizens’ jury afforded substantial time for RAPID-RT to be explained, questions to be asked and answered, and consisted of several activities – group discussions and polling - that encouraged juror reflection over the subject area. Furthermore, the design and conduct of the jury was considerably strengthened through significant PPIE embedded from the outset.

A potential limitation of this citizens’ jury relates to challenges around recruitment, with recognition that certain groups may not have been represented. Whilst the design team considered patient demographics (of those receiving radiotherapy for lung cancer at the Christie), they found ethnicity data was poorly collected. Subsequently, recruitment was sought to match a wider demographic of the GM area. Within the selected jury sample, individual-level criteria e.g. smoking status, type of lung cancer and prognosis (where a juror was a patient), was not specifically collected. Combined with this, gender was distributed unevenly amongst the selected jury, whilst all jurors felt comfortable listening and conversing in English. We acknowledge that significant disparities in lung cancer diagnosis and incidence exist compounded by risk factors such as smoking behaviour and socioeconomic status. In a large English-based study, Chen and colleagues [59] report clear evidence of how ethnic background and social circumstance influence risk of developing cancer, the type of lung cancer and how the disease develops. This considered, it is possible that varying attitudes regarding data use were not captured, and underpinning reasons that could be specific to individuals’ lived experiences, which could include for example, particular risk-averseness to data use, trust in health services and research or lack of cultural inclusivity (in corresponding communications and understanding of data use) were not explored [60]. There are also practical limitations that include the withdrawal of jurors (as encountered with the dropout of one juror between days) and the time commitment required to attend a two-day citizens’ jury. This, for example, may reduce participation from people in full-time employment or people with caring responsibilities. Given that we acknowledge limitations within our sample, the decisions reported within this article must be viewed with this consideration. To add here, we are conscious that the issue of ‘representativeness’ and generalisability are contested concepts within the design of citizens’ juries [61]. Citizen juries are informed by deliberative democratic principles that include an exchange of ideas and reasoning based on the common good rather than individual experience alone [61]. Subsequently, we aimed for inclusivity with acknowledgment that true inclusivity is difficult to achieve for some of the reasons stated. Similarly, the citizens’ jury was specifically designed for RAPID-RT and it is possible that within other contexts, attitudes towards data use may differ from what we report in this article.

In respect to the polling activities undertaken, the method was designed to elicit responses for each option rather than comparing between options. To this effect, differentiation between responses given as ‘strongly agree’ and ‘agree’ were not statistically examined, meaning that greater insight into jurors’ preferences were not available. Similarly, the results were viewed at the whole jury level meaning that when summarising the jury, particular individual views or outliers were not prioritised unless this was shared across groups or warranted further group discussion. A further potential limitation is the influence of the research team and expert witnesses on juror’s decision-making through their presence and the content of presentations. Whilst efforts were made to ensure the design of the jury was one which lay participants and advisors were comfortable with, this influence on decision-making cannot entirely be ruled out even though it forms a key component of the citizens’ jury method [34]. Where expert witnesses external to the research team could be called upon, this was done (five of the seven expert witnesses were external). A later aim of RAPID-RT is to communicate to other researchers the benefits and disadvantages of the relatively novel research approach of rapid-learning using RWD [12]. It is hoped that as this method becomes more widespread, there will be a broader pool of independent experts to call upon and present information.

## Conclusion and Next Steps

An informed opt-out approach for the use of anonymised RWD was considered most acceptable by a two-day citizens’ jury, comprising of people living with cancer, people with caring responsibilities for those living with cancer, and members of the public with an interest in health research. An informed opt-out approach was considered proportionate to the perceived ‘low-risk’ nature of the study as well as being more pro-active in normalising participation. The need to explain the use of data to patients, primarily through a consultation with a HCP followed by other communication methods, was considered necessary for purposes of transparency and to overcome concerns about potential third-party data use. The importance of patient-facing study information, traditionally associated with opt-in approaches to consent, challenges narratives that opt-out approaches to consent ordinarily involve less information provision to patients. There are important ethical considerations regarding how RWD is accessed, used, and managed. Fundamentally, processes must be in place to ensure patient trust is maintained when using such data.

A number of steps have been taken to implement the decisions and advice expressed by the citizens’ jury [12]. RAPID-RT has adopted an informed opt-out approach to take consent for the use of RWD from patients. Furthermore, several communication methods are being used to explain the use of RWD to patients, including in-person communication with a HCP, a short (two-page) PIS describing the study in plain English, together with an audio version and a short digital video that describes the study in greater detail. Providing information both in person and in forms which are able to be accessed at a time of patients’ own choosing reflects both the preference for a variety of information types, and that it is difficult to predict when patients will feel ready to engage with it. Ethical approval for RAPID-RT to recruit patients with stage I-III lung cancer receiving curative intent radiotherapy at the Christie Hospital NHS Foundation Trust was granted in February 2023 (REC reference 22/NW/0390; Sponsor: The Christie Hospital NHS Foundation Trust). Patient and clinical experience of the informed opt-out approach and these supporting forms of information are being captured through qualitative interviews. These interviews explore the interaction that takes place between patient and clinician, explore patients’ understanding of the information about the use of their data, and examine whether patients and clinicians are comfortable with the way patients’ data is used in rapid-learning studies in practice. It is possible that this will also enable the team to engage with groups that did not participate in the citizens’ jury or individuals that may opt-out of the process, and further understand different views towards consent and how data is to be used.

## Appendix 1: Set of polling questions asked to jurors over the course of the citizens’ jury

Jurors were asked to circle their preferences for each of the following questions asked. This was done at three different time points across the two days: start of the jury day one (Poll 1); close of jury day one (Poll 2); and close of jury day two (Poll 3).

**Table:** 

Citizens’ Jury					
**1. What is the most appropriate way for patients to consent to their anonymised data being used in the RAPID-RT Study?**					
**Patients should give fully informed written consent before we use patients’ anonymised data**					
Strongly Agree	Agree	Disagree	Strongly Disagree	Don’t know	Don’t understand the question
**Simplified Opt-in consent should be given before we use patients’ anonymised data**					
Strongly Agree	Agree	Disagree	Strongly Disagree	Don’t know	Don’t understand the question
**Opt-out consent is sufficient before we use patients’ anonymised data**					
Strongly Agree	Agree	Disagree	Strongly Disagree	Don’t know	Don’t understand the question
**No additional consent is needed to use patients’ anonymised data**					
Strongly Agree	Agree	Disagree	Strongly Disagree	Don’t know	Don’t understand the question
Citizens’ Jury					
**2. How do you think information about the RAPID-RT study should be delivered to our patients?**					
**In person by healthcare professional e.g. doctor or nurse**					
Strongly Agree	Agree	Disagree	Strongly Disagree	Don’t know	Don’t understand the question
**Via a patient information sheet given at time treatment is discussed**					
Strongly Agree	Agree	Disagree	Strongly Disagree	Don’t know	Don’t understand the question
**Via a flyer that explains how the data will be used**					
Strongly Agree	Agree	Disagree	Strongly Disagree	Don’t know	Don’t understand the question
**Via a poster on the walls of the waiting room/treatment room**					
Strongly Agree	Agree	Disagree	Strongly Disagree	Don’t know	Don’t understand the question
**Through a QR code that links to more information**					
Strongly Agree	Agree	Disagree	Strongly Disagree	Don’t know	Don’t understand the question
**A short online film with audio and subtitles**					
Strongly Agree	Agree	Disagree	Strongly Disagree	Don’t know	Don’t understand the question
**A recorded message that can be accessed by phone**					
Strongly Agree	Agree	Disagree	Strongly Disagree	Don’t know	Don’t understand the question

## Data Availability

The anonymised data that supports the findings of this study are available on request from the corresponding author [SD]. The data are not publicly available due to containing information that could compromise research participant privacy.
