# Correction: Exploring Consent to Use Real-World Data in Lung Cancer Radiotherapy: Decision of a Citizens’ Jury for an ‘Informed Opt-Out’ Approach

**DOI:** 10.1007/s10728-025-00532-3

**Published:** 2025-07-26

**Authors:** Arbaz Kapadi, Hannah Turner-Uaandja, Rebecca Holley, Kate Wicks, Leila Hamrang, Brian Turner, Tjeerd van Staa, Catherine Bowden, Annie Keane, Gareth Price, Corinne Faivre-Finn, David French, Caroline Sanders, Søren Holm, Sarah Devaney

**Affiliations:** 1 Manchester Centre for Health Psychology, Division of Psychology and Mental Health, School of Health Sciences, Faculty of Biology, Medicine and Health, The University of Manchester, Manchester, UK; 2 Vocal, Research and Innovation Division, Manchester University NHS Foundation Trust, Manchester, UK; 3 Department of Radiotherapy Related Research, Division of Cancer Sciences, School of Medical Sciences, Faculty of Biology, Medicine and Health, The University of Manchester, Manchester, UK; 4 RAPID-RT PPI Advisory Group, Manchester, UK; 5 Division of Informatics, Imaging and Data Science, School of Health Sciences, Centre for Health Informatics and Health Data Research UK North, The University of Manchester, Manchester, UK; 6 Centre for Social Ethics and Policy, Division of Law, School of Social Sciences, The Faculty of Humanities, The University of Manchester, Manchester, UK; 7 Department of Clinical Oncology, The Christie NHS Foundation Trust, Manchester, UK; 8 Centre for Primary Care and Health Services Research, NIHR Greater Manchester Patient Safety Research Collaboration, Division of Population Health, Health Services Research and Primary Care, School of Health Sciences, Faculty of Biology, Medicine and Health, The University of Manchester, Manchester, UK

**Correction to**:** Health Care Analysis (2025) 33:192–213** **https://doi.org/10.1007/s10728-025-00510-9**

In the original version of this article, the Acknowledgements, Author Contributions, Funding, Conflict of Interest, Ethics Approval, and Availability of Data and Material was inadvertently omitted. These missing statements are provided here.

**Acknowledgements** We would like to extend our gratitude to jurors who participated in the citizens’ jury. We would also like to thank Vocal for their central role in organising the citizens’ jury as well as all expert witnesses and citizens’ friends.

**Author Contributions **AK^1^: analysis, drafting manuscript, manuscript review and editing. SD SH: conceptualization, design of the citizens’ jury and study methodology, supervision, analysis, manuscript review and editing. GP CFF: funding acquisition, conceptualization, design of the citizens’ jury and methodology, manuscript review and editing. HTU AK^2^ LH BT: design of the citizens’ jury and study methodology, manuscript review and editing. RH KW: design of the citizens’ jury and study methodology, analysis, project administration, manuscript review and editing CB: manuscript review and editing. TvS, DF, CS: design of the citizens’ jury and study methodology**. **

**Funding** This project is funded by the National Institute for Health and Care Research (NIHR) under its Programme Grants for Applied Research (PGfAR) (Grant Reference Number NIHR202024). DF was supported by the NIHR Manchester Biomedical Research Centre (BRC) (IS-BRC-1215-20007 and NIHR203308). GP and CFF were supported by Cancer Research UK RadNet Manchester [C1994/A28701] and the NIHR Manchester BRC (NIHR203308). The development patient and public involvement and engagement (PPIE) work conducted with programme collaborators, Vocal, was supported by the NIHR Manchester BRC (NIHR203308). The views expressed are those of the author(s) and not necessarily those of the NIHR or the Department of Health and Social Care.

**Availability of Data and Material **The anonymised data that supports the findings of this study are available on request from the corresponding author [SD]. The data are not publicly available due to containing information that could compromise research participant privacy.

**Conflict of Interest** The authors declare no competing interests.

**Ethics Approval: **The citizens’ jury was a PPIE initiative. As such, no specific ethical approval for this activity was required or sought.

The original article has been corrected.

